# Knowledge, attitude and practice of dentists toward providing care to the geriatric patients

**DOI:** 10.1186/s12877-021-02343-2

**Published:** 2021-06-30

**Authors:** Bahareh Tahani, Skekoufeh Sedaghat Manesh

**Affiliations:** 1 Dental Reaserch Center, Dental Research Institute, Oral Public Health Department, School of Dentistry, Isfahan University of Medical Sciences, Isfahan, Iran; 2 Dental Students Research Center, School of Dentistry, Isfahan University of Medical Sciences, Isfahan, Iran

**Keywords:** Knowledge, Attitude, Practice, Geriatric dental care, Dentists, Iran

## Abstract

**Background:**

Tooth loss, systemic diseases and medications add to the complexity of the oral conditions in geriatric popuation, making this age group a special group in need of specific preventive and curative oral health care. Therefore, the dental teams need to be equipped with specific knowledge and skills to provide the appropriate dental care. This study was iaimed to assess the knowledge, attitude, practice and willingness of general dentists to provide dental care to geriatric patients.

**Methods:**

This cross-sectional study was conducted on 231 dentists using a questionnaire including demographic characteristics, knowledge, attitude and practice sections. The availability of the facilities for providing services to the older people in the office was also evaluated. Data were fed into SPSS-22 and analysed using descriptive statistics, t-test, Chi-squared, ANOVA and correlation coefficient tests (α = 0.05).

**Results:**

62.8% of the participants were women and their mean age was 34. 4±8. 1 years. The knowledge mean score was 13.3± 2.9 (out of 30). 86. 5% of the participants had moderate knowledge, and 2.6% displayed good knowledge. The mean score related to the attitudes toward geriatric was 55.8 ± 6. 1 (out of 85), which was not significantly different based on gender. The mean score of practice was 21. 4± 4.3. There was a significantly positive correlation between knowledge and attitude. Dentists with a higher knowledge score had moderately a more positive attitude towards the older people (*R* = 0.33, *p*_ value < 0.001). However, over 60% preferred to provide care to the young patients. Significant correlation was observed between their practice and attitude (R = 0. 2, *P*-value = 0.006). Nearly 30% of the dentists found their knowledge and experience insufficient in treating the older people with complex medical problems. 40% believed that the current dental education in dental schools did not provide adequate training in geriatric dental care.

**Conclusion:**

Although the dentists in this study had average knowledge and capacity, they mostly displayed a positive attitude towards the old. A high percentage of them were unsatisfied with the sufficiency of geriatric dental education in dental schools.

## Background

Rapid demographic changes with a growing population of the elderly have occurs around the world. Given the current trend, it is expected that older people population will triple by 2050 and reach two billion [1]. In Iran, as a developing country, the population of the geriatric people (≥65 years old) will increase from the current 8 to 22% by 2045 [2]. Therefore, countries should be prepared for the aging phenomenon and adopt appropriate policies to meet their health needs.

Getting older is associated with a higher incidence of some chronic diseases most of which have been proven to have oral manifestations that might result in limitations in chewing and swallowing [3]. On the other hand, the relationship between oral health and general health has also been indicated. Hung et al. [4]., based on the analysis of the data obtained from the 2015–2016 cycle of the National Health and Nutrition Examination Survey (NHANES), reported that out of the 10 systemic diseases investigated, six were associated with oral health outcomes including diabetes, coronary heart disease, congestive heart failure, high blood pressure, asthma and liver condition. In addition, difficulties in performing oral health self-care as a result of physical and mental disabilities can lead to poor oral health in the older people [5]. Further, lack of access to dental services as a result of financial constraints and lack of family support are other common problems that could potentially jeopardise the oral health of people over 65 [6].

Despite the fact that the edentulous rate has been decreased by 50–60% in the last 20 years in the developed countries [7], the prevalence of caries and tooth loss is high in the developing countries. It has been estimated that about 52% of people over 65 are edentulous in Iran. This is even as high as 80% in some provinces [8]. In general, oral conditions are usually complicated in older people due to systemic diseases and medications and make this age group a special group in need of specific preventive and curative oral health care. The dental teams therefore need to be equipped with specific knowledge and skills to provide the appropriate dental care [9]. However, studies conducted on dentists in different parts of the world have indicated that their knowledge is usually unsatisfactory [10–12]. According to a survey, nearly 20% of the graduated dental students in Belgium reported that they were not well-prepared to provide care to the older people due to lack of enough knowledge [13].

Formation of attitudes towards older people is also a key component in the development of professional behaviors and practical patterns of dental students [14]. Attitudes are defined as learned predispositions that steer the people’s responses in a consistently favourable or unfavourable manner. However, dentists and senior students sometimes do not display positive attitudes towards providing dental care to the older people [15]. Kuthy et al. showed that about 37% of senior dental students were unwilling to provide care in future to the elderly patients [16]. This finding was also reported in another study conducted by Major et al., showing that even anticipated willingness to treat became more negative towards the elderly patients as students progressed through their predoctoral education [17].

Accreditation standards of dental education in dental schools require that students be sensitized to underserved populations’ needs. They should know about different care delivery models that can address barriers to care in order to provide appropriate treatments. In US dental schools, different courses, mostly in the form of community-based education, have been developed to reach these goals [18]. However, some studies have revealed that most dental schools in Iran have not sufficiently covered geriatric dental education in their curriculum [19]. Given the growing population of the older people in the country and the scarcity of data about the dentists’ preparation status, this study aimed to assess the knowledge, attitude and practice of general dentists in regard to geriatric dentistry, as well as evaluating the availability of the required care facilities in the dental settings.

## Methods

This cross-sectional study was approved by the Ethics Committee of the Vice Chancellor for Research at Isfahan University of Medical Sciences; it was registered under the codes 397,232 and IR.MUI.Research.REC.1397.364. Participation in this study was voluntary, and the informed consent was gained.

### Sampling

Participants included general dentists working in dental clinics and private offices with at least 1 year of experience. We used the sampling formula for qualitative variables like proportion($n=\frac{{\left(z1-\frac{\alpha }{2}\right)}^2\cdotp p\left(1-p\right)}{d^2}$), where Z_1-a/_ 2 = standard normal variate (at 5% type 1error (*P* < 0.05) it is 1.96) and p = Expected proportion in population based on previous studies or pilot studies. d = absolute error or precision and has to be decided by researcher [20]. In our study, assuming an α (type I error) of 0.05 and taking into account the percentage of dentists with good knowledge (11%) about geriatric dentistry in other similar study [10] and the precision of 5%, the sample size was calculated to be 150. Considering the design effect of 1.5 of cluster sampling and considering the probable sample loss, it was calculated to recruit 240 samples.

According to the map of Isfahan city, from 14 municipal regions, 6 ones were chosen randomly using random digit numbers. List of dentists in each region was obtained from the Medical Council and in each of the selected regions, 40 dentists were chosen based on the systematic random selection including 25 dentists working in dental clinics and 15 from those working in private offices in each selected region. The study was carried out from March to June, 2019, in Isfahan which is the country’s second biggest city after the capital (Tehran). It has two dental school and the second highest concentration of dentists per population after the capital. The total number of general dentists was about 1500 according to the Isfahan Medical Council in 2019.

### Data collection tools

A self-administrated questionnaire was used to collect the data. The first part of the questionnaire included demographic information such as gender, age and frequency of visiting different age groups in offices or clinics; it also included questions about passing a geriatric dentistry course at dental schools. The second part of the questionnaire comprised 27 questions asking about the dentists’ knowledge (True / False / Don’t Know), with scores in the range of 0–30. Questions were adapted from Hatami et al. [21]. The themes of the knowledge section included the normal aging of the oral cavity, common oral conditions in the older people, social aspects of aging, and dental care modifications for the older adults. Reliability and validity of the knowledge questions were assures previously [21] .

For the attitude part of the questionnaire, 17 questions were selected based on a 5-point Likert scale ranging from strongly disagree = 1 to strongly agree = 5. Items were developed based on the Persian version of the Geriatric Attitudes Scale [22], which had been translated and validated previously [21]. The original Geriatric Attitude Scale has 14 questions; these were all included in the questionnaire applied the current study, except the statement of “older people act too slow for modern society”, which was replaced with “I understand the problems of ageing including physical and mental limitations”. Also, three other questions related to providing care in dental offices were added; these included “welcoming the admission of older people in dental office, whether older people followed the dental advice provided to them, and if providing dental care to the older takes more time than for the young”.

The fourth section, which contained 7 questions about the practice scheme, included the self-perceived ability of the dentists in providing preventive and treatment plans, management of the older people’s emergencies, and communication and emotional skills. The items were scored based on a five-point Likert scale (ranging from 5 = completely agree to 1 = completely disagree). The desired level of practice was considered to be 23 (70% of the range based on the subjective judgment of the principal investigators (PIs) of the current study and the literature recommendations) [23].

The availability of the facilities for providing services to the older people in the office was also evaluated based on a checklist [24]. The items of the checklist were about the availability of accommodations to reduce the risk of falling and the vision impairment (appropriate flooring), the presence of a suitable elevator, the presence of ramps on the paths with wall handles, and the availability of sufficient wheelchairs in dental settings.

The questionnaires were distributed among the dentists at the beginning of their workday by one of the PIs. They were asked to complete the questionnaires on the same day if they were willing and return it back. Descriptions about the objective of the survey, contact number of the researchers, code of ethics, the assurance about the anonymity of responses and the voluntarily participation of them were provided on the cover page of the questionnaires. It was also emphasized to answer the questions truthfully without consultation with other colleagues. Sampling was continued till the predicted sample size was achieved.

### Statistical analysis

Data were fed into the SPSS software (IBM SPSS Statistics for Windows, Version 22.0. Armonk, NY: IBM Corp) and analysed by descriptive and analytical statistics. The mean scores of knowledge and attitude were calculated. Based on the total scores obtained, the relationship between knowledge and attitude mean scores and their correlation with age and mean work experience were analysed using Pearson correlation coefficients. ANOVA test was used to assess the differences of knowledge and attitude mean scores based on the frequency of the older patients admitted to office or clinic, as well as passing a geriatric course at university and having relationship with their grandparents. Differences based on gender was analysed using T-test. *P* ≤ 0.05 was considered significant for all statistical analyses and was adjusted in multi-comparisons based on the number of comparisons.

## Results

A total of 231 completed questionnaires (response rate = 88%) were obtained. Mean of age was 34.4 ± 8.1 (23–71 years) and 62.8% (*n* = 145) were female. Mean work experience was 9. 2 ± 6.8 years (42–1 years). The percentage of the patients in different age groups visited by the dentists during the past month showed that the most frequent age group was the 18–40 year group (40%). The frequency of patients in age group of 65 and older was about 13%.

Further, 54% of the dentists (*n* = 117) reported that either their father or mother was old and 45% (*n* = 101) stated they had close relationships with their grandparents (Table 1). Dentists’ preferences in providing services to different age groups are shown in Table 1. Accordingly, 64% of the dentists preferred to provide services to the adults aged 25–44 years and 5% of them were interested in providing services to the older people over 65.

**Table 1:** Demographics and work characteristics of the dentists

	Frequency	Percentage
Gender		
Male	86	37.2
Female	145	62.8
Time since graduation		
< 5 years	56	35.9
5–15 years	65	41.7
Over 15 years	35	22.4
Percentage of the patients visited in the last month in the age group over 65		
< 15%	116	72.5
15–30%	30	18.8
> 30%	14	8.8
Having an old father/mother		
yes	117	54.2
Relationships with their grandparents		
Close relationship	101	44.9
Not so close	18	8
They are not alive	106	47.1
Preference of the dentists in providing care to different age groups		
1–4 years	4	1.8
5–12 years	43	18.9
13–18 years	46	20.3
19–24 years	70	30.8
25–44 years	144	63.4
45–65 years	45	19.8
Over 65 years	12	5.3
illingness to attend continuous training courses on geriatric dentistry		
Highly willing	34	15
Almost willing	109	48
Almost unwilling	65	28.6
Highly unwilling	19	8.4

### Knowledge status

The mean score of knowledge was 13.3 ± 2.9 (out of 30). Considering the cut-off points 0–9, 10–19 and 19–27 as poor, moderate and good, respectively, it was shown that 2.6% (*n* = 6) had good knowledge and 10.8% (*n* = 24) had poor knowledge. Pearson correlation test showed no significant correlation between knowledge and duration of work experience or age. Table 2 demonstrates the comparison of knowledge mean scores among dentists based on their demographics and work characteristics. The mean score of knowledge was significantly different among dentists based on the percentage of their patients in the age group 65 and over (ANOVA, *P* = .02). However, based on the Post-hoc Tukey analysis and adopting the Bonferroni adjustment, comparisons between groups was not significant. There was also no significant difference between dentists’ mean scores based on their gender (T-test).

**Table 2:** Comparison of the mean scores of dentists’ knowledge and attitude based on their demographics and work characteristics

	Mean of Knowledge	SD	*P*-value	Mean of attitude	SD	*P*-value
Gender						
Male	13.5	2.8	0.2*	55.3	6.3	0.3*
Female	13.3	3. 1		56.1	5.9	
Time since graduation						
< 5 years	13.9	2.5	0.2**	55.7	5. 4	0.6**
5–15 years	13.3	3. 1		55.6	6.7	
Over 15 years	14.3	2. 1		56.9	6.4	
Percentage of over 65-year patients visited per month						
< 15%	13.9	2.6	0.02**	56.0	6.4	0.2**
15–30%	12.9	3.5		54.4	6.8	
> 30%	12.0	3.7		53.2	5.9	
Having an old father/mother						
yes	13.1	3.1	0.08*	56.1	6.2	0.6*
no	13.8	2.8		55.6	6.1	
Relationships with their grandparents						
Close relationship	13.1	2.8	0.08**	55.1	5.8	0.018**
Not so close	12.5	3.0		53.5	5.4	
They are not alive	13.9	2.9		57.1	5.9	
Willingness to attend continuous training courses on geriatric dentistry						
Highly unwilling	13.1	2.2	0.3**	50.1	4.8	< 0.001**
Almost unwilling	13.1	2.7		53.7	6.3	
Almost willing	13.2	3.2		57.5	5.2	
Highly willing	14.1	2.5		56.7	6.1	

Note- *-T-test, **-ANOVA test

*Attitude Status*

**Fig. 1:**
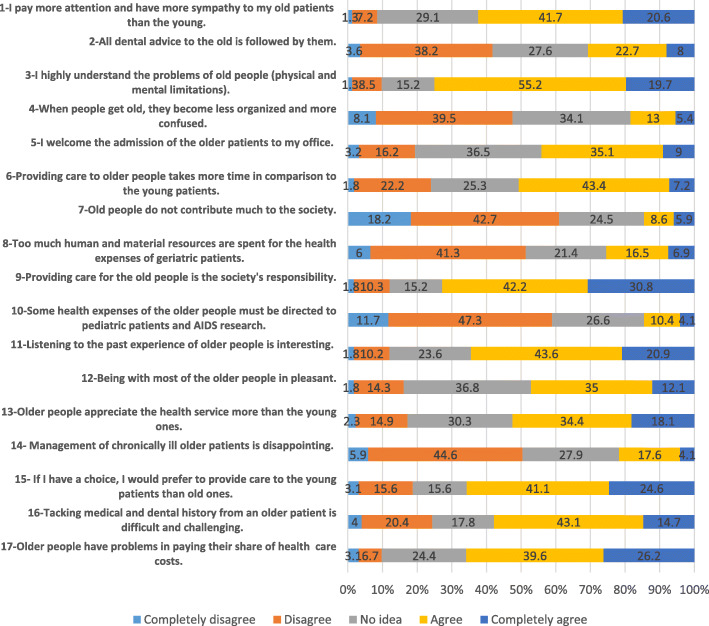
Attitude of the general dentists towards providing dental care to the old people

The attitudes of the dentists towards the older people are presented in Fig. 1. About 67% of them believed that the older people were not able to pay their share of dental care costs. Also, almost 60% thought it was difficult to gain a medical and dental history of them; further, over 60% preferred to provide care to the young rather than the old. A high percentage of dentists (73%) believed (agree or completely agree) that looking after the older people was the society’s responsibility. Moreover, a large number of them (75%) reported that they understood the physical and mental problems of this age group and 30% held the view that the advice given to the older people was fully taken by them. Also, 52% of the dentists believed that older people appreciated the health service more than the young people.

Recoding the negative attitude statements and summing all scores, the dentists’ mean score of attitude was 55.8 ± 6.1 (41–70). Higher scores meant a more positive attitude toward geriatric populations. Regarding the probable scoring interval of 17–85 for the attitude section, dentists’ attitude toward geriatric patients was higher than the average of 34 and tended to the higher scores. Table 2 presents the comparison of attitude scores among dentists based on their demographics and work characteristics. The mean score of attitude was not significantly different based on gender or age of dentists. The dentists who were more willing to attend geriatric educational courses (ANOVA, *P*-value< 0.001) and those whose grandparents had passed away had a significantly higher positive attitude (ANOVA, *P*-value = 0.018). However, results of Post-hoc Tukey (*p* = 0.04) analysis and considering the adjusting effect of Bonferroni for multiple comparisons (α/3) indicated that the later finding was not significant any more. For the relationship between mean scores of knowledge and dentists’ willingness to attend educational courses, the post-hoc analysis adjusted based on the number of comparisons (α/6) reaved that those who were highly willing gained higher attitude scores in compare with those who were not willing at all (*p* = 0.003). Dentists who were almost willing to attend such courses showed higher scores in attitude in comparison with those who were highly unwilling (*p* < 0.001) and almost unwilling (*p* = .001).

There was also a significant relationship between the dentists’ knowledge and attitude mean scores; therefore, the dentists with a higher knowledge score had a more positive attitude towards the older people (R = 0.33, *p*_ value < 0.001, Pearson correlation test).

### Practice status

**Fig. 2:**
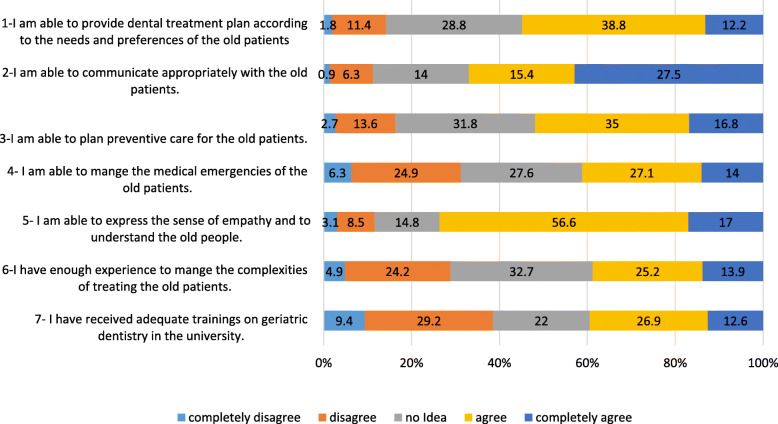
Self-reported ability of the dentists towards the provision of dental care to the old people

The results of the dentists’ self-reported ability in dealing with the older people’s problems are shown in Fig. 2. According to the results, more than half of the dentists found themselves capable of providing the appropriate treatment to the older people and about 40% were satisfied with their ability to communicate. Nearly 30% of the dentists found their knowledge and experience insufficient in treating the older people with complex medical problems and about 40% believed that the current dental education in dental schools did not provide adequate training in geriatric care. By summing the scores of responses to the questions 1–6 of the ability section, the mean score of the practice was 21.2 ± 4.3 (9–30). There was no significant correlation between the dentists’ knowledge and practice. A significant correlation was observed between their practice and attitude (R = 0.2, *P*-value = 0.006).

Availability of equipment for the ease of access revealed that 54% of dental offices/clinics had adequate lifts. Only about 20% of them had ramps and wall hangings and almost 24% had good flooring.

## Discussion

The results of the current study revealed that only about 3% had good knowledge, and most of them had a moderately positive attitude about older people. There was a significantly weak positive relationship between the dentists’ knowledge and attitude, and between their attitude and practice. However, only 5% of the dentists were interested in providing services to the older people and a large number of them believed that their academic dental education had covered the geriatric dentistry inadequately.

In agreement with the findings of our study, in a survey conducted by Moreira et al. [25] in Brazil, most of the dentists had moderate knowledge and attitude towards the older people. A significant relationship between gender and attitude was reported in the study conducted by Bots-VantSpijker et al. in Netherlands and Belgium [26] where women showed a more positive attitude. They argued that higher attitude scores in women could be due to their higher level of empathy and emotions. However, the difference was not significant in our study.

Alaee et al. [10] also repored that the majority of dentists were not well aware of the geriatric dentistry (88.5 and 11.5% had poor and moderate knowledge, respectively). However, in our study, the majority of the participants had moderate knowledge and 10.8% reported poor knowledge of geriatric dentistry. This discrepancy can be partly due to the number and type of the questions posed. Hatami et al. [21] showed that the majority of students had low-to-moderate knowledge of and attitude towards geriatric dentistry.

In our study, about 10% of the dentists reported that more than one third of their accepted patients in the last month were in the age group of 65 and above. In a study conducted by Bots-VantSpijker et al. [26] on dentists in Europe, 25 and 32% of the dentists working in the Netherlands and Belgium, respectively, reported that 30–100% of their patients belonged to the age group of ≥65 [26]. This can be partly explained by the differences in the level of geriatric education between the dental schools of Iran and Europe. It is known that acquiring sufficient clinical experience in dental schools makes the dentists feel clinically confident and comfortable in the treatment of the older patients [27].

Preshaw et al. [28] evaluated the education status of geriatric dentistry in some European dental schools and revealed that the majority (93%) of universities had modules and special courses dedicated to the old people. In our study, a high percentage of dentists found the present training in dental schools inadequate. In addition, a study addressing the dental schools of Iran [19] revealed that their claim was almost true and the courses and educational time allocated to geriatric education were not sufficient. In accordance with the geriatric dental education, eight schools from the 11 (72.72%) main schools which had been established at least 6 years earlier had an elderly dental education module. Although all of them mentioned considering some hours of training, only one school (12.5%) had a group seminar or occasional lectures. Among these eight schools, four (50%) had clinical education in the field of geriatric dentistry. However, none of the schools had a separate department for geriatric dentistry or a specialized clinic for the elderly and a referral system devoted to referring the older patients from hospitals to faculties.

Although it has been shown that knowledge of aging can be learned easily by dental students throughout academic courses, the link between knowledge gain and attitude shift cannot be made directly [29]. Considering correlation coefficients < 0.35 as low or weak correlations, 0.36 to 0.67 as modest or moderate correlations, and 0.68 to 1.0 as strong or high correlations [30], in our study also there was a weak (although significant) correlation between knowledge and attitude. Different discipline-specific interventions, including aging-awareness training, multi-modal interventions (e.g., didactic lectures, group activities, simulations, and mentorship), clinical geriatric rotations, senior mentoring programs and infusion of aging content into the curriculum, have been utilized, all aiming at shaping, changing and influencing attitudes positively [31]. Positive personal experience with older people and societal influence have been suggested as the main factors involved in predicting the attitudes towards this age group [32]. Professional socialization including exposure to the faculty members who hold positive attitudes towards the older adults, can play an important role in the formation of positive attitudes towards the older adults among dental students [33]. However, in a survey conducted in Iowa university by Major et al. [17], it was revealed that many significant changes had occurred in the feelings towards treatment and willingness to treat specific underserved populations including the elderly, five years after graduation compared to their first year of dental school when they were more negative towards treating low-income patients and the frail elderly. They also suggested that some variables, in addition to exposure to patients, might shape the students’ willingness to treat the underserved populations.

Regarding the barriers to access to dental care, Borreani et al. [34] categorized cost, fear, availability, accessibility and characteristics of the dentist as the main five active barriers to dental care in the older people. In agreement with the findings of Borreani et al. [34], the barriers felt by the dentists in providing care to the older people in the present study included the old people’s inability to pay the costs, difficulty in getting their history, and problems in the communication with them.

In Iran, as a developing country, the state, the insurance system (public and commercial), and the private sector are the three main health care delivery sectors [35]. The Ministry of Health and Medical Education (MOH) is the main provider of oral health care (OHC) services in the state sector. There are, however, limited public dental clinics (PDCs) in the country (about 1942) where the paid dentists provide primary OHC services (extraction, fluoride varnish application, restorative treatments, scaling and root planning) mostly to the children under 12 and pregnant and nursing women [35]. However, the main sector in Iran is the private sector comprising about 90% of the oral health delivery system. Based on a survey of Iranian population conducted in 2017, it was reported that about 60% of the older people (≥65 years old) were covered by public insurance and 10% had no insurance at all [36]. About 30% of the older people were able to use dental care services (at least one dental visit per year).

Currently, in most high-income countries, some form of dental care is provided to the children and youth, but the coverage of the dental care available to the older population varies to some extent. In Canada, Australia and Italy, which have the most publicly-funded health care systems, dental care is predominantly privately funded with a combination of out-of-pocket payments and private insurance [37]. Considering the income threshold, it has been estimated that less than half of the population of adults over the age of 65 in Australia can be eligible for any public coverage (Commonwealth Seniors Health Card and Pensioner Concession Card programs), covering some of their dental care costs. Also, 46% of residents over 65 and older have some level of private health insurance that can cover dental care [38].

In addition to the limited public coverage of dental care in most countries and lack of public coverage for some complicated and high-cost services such as crowns, bridges and dentures, supply constraints within public dental care clinics and the high demand for dental care have also led to long waiting times for dental care. In order to facilitate the older people’s access to dental care, different strategies need to be taken into consideration, including individual measures (e.g. persuading to receive dental care in case of need), system changes (e.g. cost reduction, improved information provision, and timely and proper patient management), and social issues (e.g. solving the isolation and loneliness problems). It seems therefore that system and social changes are more important than personal actions among the older people [34].

### Limitations

In Iran, about 90% of dental care is provided by the private sector. In Isfahan city, there are just 3 public dental clinics, one of which was covered in our sampling geographic areas. Although most of the dentists are working in the private sector, we did not ask them about their work place which might have influenced their responses in regard to their control over the working set-ups and attitude section. Besides, we did not evaluate the existence of the required facilities in dental settings by observation and it was reported by the dentists themselves.

Another limitation might be the proportion of female to male dentists in our participants. Although there is no updated data regarding the current proportion in the country, according to the available data on dental students, about 56–67% of them are female [39, 40]. The higher proportion of female dentists in our study (62%) might have influenced the frequency of responses, especially in regard to the attitude section. However, it seems that the gender proportion of our participants is similar to the proportion of dentists at the country level that besides the similarity of the national educational curriculum educated in dental schools, might assist in assuring the generalizability of our findings.

## Conclusion

The dentists in our study had moderate knowledge and ability in dealing with the older people’s dental problems. However, the majority of them had a positive attitude toward the old. Therefore, promoting geriatric education in dental schools, especially clinical education, should be on the agenda of dental education policy-makers. In addition, running continuous courses and conferences in this regard, including geriatric physiological changes, communication skills and care management principles, can be very effective for the graduated dentists. Moreover, given the lack of facilities and standards required to provide services to the older people, it is necessary to inform dentists about the standards and to emphasize the need for compliance with them by establishing appropriate regulatory policies.

## Data Availability

The datasets used and/or analysed during the current study are available from the corresponding author on reasonable request.
